# Lung cancer mortality in a cohort of UK cotton workers: an extended follow-up

**DOI:** 10.1038/bjc.2011.312

**Published:** 2011-08-16

**Authors:** D M McElvenny, M A Hurley, V Lenters, D Heederik, S Wilkinson, D Coggon

**Affiliations:** 1 School of Health, University of Central Lancashire, Preston, Lancashire, PR1 2HE, UK; 2 Statistics and Health Limited, 12 Turfnell Way, Worsley, Manchester, M28 2PZ, UK; 3 Institute for Risk Assessment Sciences, Division of Environmental Epidemiology, Utrecht University, Utrecht, NL-3508 TD, The Netherlands; 4 Epidemiology Group, Health & Safety Executive, Bootle, Merseyside, L20 7HS, UK; 5 MRC Lifecourse Epidemiology Unit, University of Southampton, Southampton, SO16 6YD, UK

**Keywords:** lung cancer, cotton, endotoxin

## Abstract

**Background:**

A recent systematic review and meta-analysis suggested that occupational exposure to endotoxins protects against lung cancer. To explore this hypothesis further, the follow-up of mortality of a cohort of 3551 workers, who were employed in the British cotton industry during 1966–1971, was extended by 23 years.

**Methods:**

Subjects had originally been recruited to a survey of respiratory disease, which collected information about occupation and smoking habits. Cumulative exposures to endotoxins were estimated from data on endotoxin levels by work areas in cotton mills. Risks of lung cancer were estimated using survival modelling.

**Results:**

During follow-up, 2018 deaths were recorded before the age of 90 years, including 128 deaths from lung cancer. After adjustment for smoking, hazard ratios (95% confidence intervals) for cumulative endotoxin exposures of ⩽30 000, >30 000 and ⩽200 000, >200 000 and ⩽400 000, >400 000 and ⩽600 000 and >600 000 endotoxin units (EU) m^−3^ years were 1, 0.8 (0.5–1.6), 0.7 (0.4–1.3), 0.6 (0.3–1.0) and 0.5 (0.3–0.9), respectively (*P* for trend=0.005).

**Conclusion:**

Our findings strengthen the evidence that occupational exposure to endotoxins protects against lung cancer, and suggest that the effect depends on cumulative dose and persists after exposure ceases.

Endotoxins are the lipopolysaccharide component of the outer membrane of Gram-negative bacteria, and can be released to the environment during cell replication and lysis (Liebers *et al*, 2008). They are ubiquitous in indoor and outdoor environments, but the highest exposures occur in certain occupational settings such as agriculture and cotton textile mills (Liebers *et al*, 2006). Exposure occurs primarily through inhalation of airborne endotoxin present in organic dusts (Liebers *et al*, 2008).

The noncancer adverse health effects of acute and chronic exposure to endotoxins are well recognised. They include localised neutrophilic inflammation leading to respiratory symptoms and lung function decrements, and at very high doses, systemic inflammation leading to the organic dust toxic syndrome (Rylander, 2002). Endotoxins and their active component, lipid A, have exhibited antitumoural effects in animal models and have shown some encouraging results in phase I and phase II clinical trials (Reisser *et al*, 2002). A recent systematic review and meta-analysis of lung cancer risk in cotton textile workers found a significantly low summary relative risk (Lenters *et al*, 2010). Moreover, the relative risk was <1.0 for most subgroups of studies distinguished according to study time period, study design, whether the outcome was mortality or morbidity, whether or not analysis included adjustment for smoking, geographical area and subjects’ sex. Two studies in the systematic review provided quantitative estimates of endotoxin exposure and these indicated a dose-dependent protective effect against lung cancer risk (Astrakianakis *et al*, 2007; Kuzmickiene and Stukonis, 2007). Findings to date on endotoxin exposure and risk of malignancies other than lung cancer have been limited and inconsistent (Lundin and Checkoway, 2009).

One of the studies included in the recent meta-analysis of cotton textile workers focussed on a British cohort in which there was a statistically significant deficit of lung cancer for men and women combined among workers with ⩾30 years of service in the cotton industry (standardised mortality ratio (SMR)=64, 95% confidence interval (CI) 40–97, 22 deaths) (Hodgson and Jones, 1990). To explore further the possible protective effect of endotoxins in cotton dust on the risk of lung and other cancers, we updated the follow-up of this cohort, which had originally been assembled in the late 1960s and early 1970s. An important strength of the study was its data on potential confounding factors, and especially on cigarette smoking. We extended follow-up by 23 years, during which time some 1500 additional deaths were expected.

## Materials and methods

### Study population

Details of how the cohort was assembled have been published (Fox *et al*, 1973a, 1973b; Hodgson and Jones, 1990). Subjects were originally recruited into a study of respiratory illness in a sample of 52 mills that spun coarse (39 mills), medium (11 mills) or fine (2 mills) cotton. Most of the mills were located in Lancashire (48), but two were in Yorkshire and two in Scotland. The mills were visited by a research team on two occasions between 1966 and 1970, and workers were eligible for the study if they were employed at a mill at the time of a visit, and were Caucasian in origin. Of the total population eligible for inclusion, ∼10% declined to participate. Between 1971 and 1974, many workers were visited again either at work or at their home address but no new subjects were recruited to the study.

At the time of recruitment, workers were interviewed, and a modified version of the UK Medical Research Council questionnaire on respiratory symptoms (Roach and Schilling, 1960) was used to collect data on symptoms and on factors that might influence their risk of respiratory disease. Among other things, participants were asked about their current job (job title and whether they worked full time or part time); when they had first worked in the cotton industry; their smoking habits (classified as nonsmoker, former smoker, and for current smokers according to the number of cigarettes smoked per day); and whether they had a cough or brought up phlegm on most days for at least 3 months of the year (Fox *et al*, 1973a). In addition, spirometry was performed, and temperature-corrected measurements of forced expiratory volume in 1 s (FEV_1_) were expressed as a percentage of predicted values derived from Cotes’ equations (Cotes, 1968), taking into account sex, age and height (no suitable reference values were available for lung function in people of non-Caucasian ethnicity in the United Kingdom at that time – hence their exclusion).

Where participants had left the industry at the time of a subsequent visit by the research team (21% of subjects), their leaving date was recorded. Total duration of employment as a cotton worker was estimated from the year in which they first entered the industry and their leaving date, if recorded, with adjustment in a few cases for known periods of absence. Where the worker was still employed at the time of the last mill visit, it was assumed that he/she remained in the industry for one further year after that visit (British cotton mills were closing rapidly in the mid-1970s).

### Endotoxin exposure assessment

From their job title at the time of recruitment, subjects were assigned to three work areas as in previous analyses (Fox *et al*, 1973a, 1973b) –‘opening’ (openers in the cotton chamber), ‘carding’ (carder, lap carrier, stripper and grinder, laptender, card tender, waste devil hole attendant, maintenance engineer) and ‘ring room/winding’ (spinner overlooker, spinner doffer, doubler, winder and beamer pirn winder). A simple job-exposure matrix was then created, in which airborne endotoxin concentrations were assigned to the three work areas, according to whether the mill spun predominantly coarse/medium or fine cotton.

To determine endotoxin concentrations, a literature search was performed, and the authors of relevant papers were contacted, to seek quantitative data, both from the United Kingdom and elsewhere, on measured endotoxin levels in the cotton textile industry and parallel measurements of endotoxin and cotton dust. Raw endotoxin data were obtained for cotton mills in Lancashire (Niven, 1993; Simpson *et al*, 1999; Fletcher, 2003) and from other countries (China, Turkey and Germany; Astrakianakis *et al*, 2006; Oldenburg *et al*, 2007; Bakirci *et al*, 2007). Additional published data on historic measurements in the UK mills were considered (Roach and Schilling, 1960; Molyneux and Tombleson, 1970; Berry *et al*, 1973; Cinkotai *et al*, 1988; Fishwick *et al*, 1994). Endotoxin levels expressed as ng m^−3^ were multiplied by 10 to approximate values in endotoxin units (EU) m^−3^. The data corresponding to each of the three work areas of interest were collected, and median, arithmetic mean and geometric mean values were calculated from the raw data and extracted from other published data, when possible. Two of the team (VL and DH) then derived expert-based ‘(best) estimates’ of average endotoxin levels by work area and type of mill, taking account of all available data, but giving greater weight to those from surveys performed in the United Kingdom (Niven, 1993; Simpson *et al*, 1999; Fletcher, 2003) (Table 1).

Individuals were assigned cumulative exposures to endotoxins (in EU m^−3^ years) calculated as the product of the estimated airborne concentration for the area in which they worked at the time of recruitment and their estimated duration of employment in the cotton industry – that is, with the assumption that the person had worked in the same area throughout his/her career. An adjustment factor of 0.5 was applied to the employment times of part-time workers. Where a worker's job title was unrecorded (*n*=179), endotoxin exposure could not be determined, and these individuals were therefore excluded from analyses relating to endotoxin exposure.

### Follow-up

The cohort was followed for mortality to the end of 2007 through the National Health Service Central Register (NHSCR). Deaths were coded according to the eighth (ICD-8), ninth (ICD-9) or tenth (ICD-10) revisions of the International Classification of Diseases (ICD), depending on the year of death. The Supplementary Table 1 shows ICD codes for the disease categories that were analysed. These disease categories were chosen *a priori* on the basis of findings from earlier cohort studies of cotton textile workers (Henderson and Enterline, 1973; Wernli *et al*, 2003, 2006; Kuzmickiene *et al*, 2004; De Roos *et al*, 2005; Chang *et al*, 2006; Gold *et al*, 2006; Wong *et al*, 2006; Li *et al*, 2006a, 2006b; Astrakianakis *et al*, 2007; Ray *et al*, 2007). Following checks against original paper records and data from the NHSCR, the number of workers whom we classed as traced differed slightly from that in the analysis that had been reported previously for the cohort (Hodgson and Jones, 1990). Sixteen workers from the original analysis were excluded because of doubtful trace information, and lost medical examination data were found in the paper records for 17 workers who had been traced. Thus, the overall cohort size was one larger than in the earlier analysis.

### Statistical analysis

Person-years analyses were conducted using the software system R (Venables and Smith, 2010) to derive SMRs and their 95% CIs. Expected numbers of deaths were calculated from national rates for England and Wales in 5-year age bands and single calendar year periods. Scottish rates were not used for the Scottish mills as there were only two of them. In addition, the SMRs were modelled within a survival modelling framework, in which it was assumed that deaths occurred at random but with a hazard of occurrence that varied over time and was specific to the individual worker (Tom and Farewell, 2009).

Follow-up began at the date of entry to the study (i.e., first medical examination). For the main analyses, workers then contributed person-years at risk until the earliest of (1) date of death, (2) loss to follow-up (mainly through emigration), (3) 31 December 2007 or (4) age 90 years.

### Ethical considerations

Ethical approval was provided by the Faculty of Health Ethics Committee at the University of Central Lancashire. The Patient Information Advisory Group of the Department of Health exempted the investigators from having retrospectively to obtain individual informed consent to inclusion in the study.

## Results

From a total cohort of 3551 workers for whom data from at least one medical examination were available, 3459 (97.4%) were successfully traced at NHSCR (Table 2). Most had worked in the cotton industry for >20 years, the earliest such employment being in 1906. The most common area of work was carding. By the end of follow-up, 2159 cohort members had died, 141 after reaching 90 years of age. In all, 44 men (2.8%) and 46 women (2.4%) were lost to follow-up (2 of the women after age 90). The total number of person-years of follow-up was 99 135.

Table 3 summarises the mortality of the cohort in comparison with the general population of England and Wales. Total mortality was significantly higher than expected (SMR=105, 95% CI: 100–109, 2018 deaths), largely because of high rates of circulatory and respiratory disease. Total cancer mortality was within the expected range (SMR=99, 95% CI: 91–108, 515 deaths), as was that from lung cancer (SMR=99, 95% CI: 82–116, 128 deaths).

Table 4 shows SMRs for lung cancer according to smoking habits and estimated duration of work in the cotton industry. In both sexes, mortality from lung cancer was highest in moderate/heavy smokers and lowest in lifelong nonsmokers. Moreover, within both light and moderate/heavy smokers, the risk of lung cancer tended to decline with longer duration of employment in the cotton industry, the trend being clearest in the analyses for both sexes combined.

The hazard ratio for death from lung cancer was modelled according to estimated cumulative exposure to endotoxins (classified to approximate fifths of its distribution in the cohort as a whole) while controlling for smoking habits (Table 5). Risk of lung cancer tended to decrease with higher cumulative exposure to endotoxins, the trend being statistically significant in men (*P*=0.05) and in both sexes combined (*P*=0.005). Among men and women combined, the overall hazard ratio in the highest relative to the lowest exposure category was 0.5 (95% CI: 0.3–0.9). When risk estimates were adjusted also for FEV_1_ (% predicted) and report of cough or phlegm at entry to the study, the trends in relation to endotoxin exposure were stronger, with an overall hazard ratio of 0.4 (95% CI: 0.2–0.8) for the highest relative to the lowest endotoxin category. No such trends were apparent in similar analyses for the combination of all cancers other than lung cancer (data not shown).

**Figure 1:**
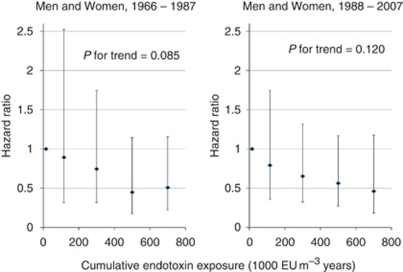
Modelled risk of death from lung cancer during 1966–1987 and 1988–2007 by estimated cumulative exposure to endotoxins. All risk estimates are adjusted for smoking habits classified as in Table 5. Bars represent 95% confidence intervals.

Figure 1 shows the relation of endotoxin exposure to lung cancer mortality earlier and later in the period of follow-up. Again, risk estimates are adjusted for smoking habits. The decline in risk of lung cancer with increasing cumulative exposure to endotoxins was clearly apparent after 1987 as well as earlier in follow-up.

## Discussion

Our study adds further weight to the evidence for a protective effect of endotoxins against lung cancer. Moreover, it suggests that protection is a function of cumulative dose, and that it continues for >10 years after cessation of exposure. In contrast, we found no evidence for protective effects against other types of cancer, indicating that the underlying biological mechanism is likely to be local rather than systemic.

Although our analysis benefited from high rates of follow-up over a prolonged period in workers with relatively high exposures to endotoxins, and also from good information on smoking habits, it had important limitations. In particular, occupational histories were incomplete, and an assumption had to be made that each subject had worked in the same mill area from the time that he/she first entered the cotton industry. The average time between the first and second examinations was 3.4 years and 95% of workers who had a job category at both examinations had identical job categories, suggesting that at least towards the end of follow-up, job mobility was very low. In addition, it was assumed that a worker's employment in the industry ended 1 year after his/her last clinical examination for workers whose leaving date had not been recorded. The last approach, which was also adopted in an earlier analysis (Hodgson and Jones, 1990), seemed reasonable as: (1) the intervals between successive visits by the research team were in the order of only 2 to 3 years, and (2) the UK cotton industry was in rapid decline by 1974 (the year of the last visit to a worker) with few mills surviving long beyond this (Hodgson and Jones, 1990).

There were also uncertainties in the retrospective quantitative assessment of endotoxin exposure levels. No direct measurements of endotoxins were available for the mills studied. Dust measurements had been made at a subgroup of 11 participating mills at around the time of the clinical examinations, using high-volume sampling over long averaging times (Fox *et al*, 1973a, 1973b). However, the data generated were too sparse and too variable for generalisation to the other 41 mills. Nor were there data on potential determinants of dust concentrations, which might have enabled modelling of levels in other mills. Thus, it was not feasible to estimate endotoxin levels by combining the cohort-specific data on dust levels with findings from other studies that had measured dust and endotoxins in parallel.

Instead, endotoxin concentrations had to be extrapolated from measurements (mostly fairly recent) made elsewhere, including in other countries. No account could be taken of the source of the cotton that was handled (endotoxin levels vary by country of origin; Lane *et al*, 2004), and there were limited data on time trends in cotton dust levels over time.

However, it seems unlikely that misclassification of exposure would have led to a spurious inverse relation to the risk of lung cancer. If anything, the misclassification would be expected to obscure true associations (Armstrong, 1998; Heederik and Attfield, 2000). Notably, the relationship between endotoxins and lung cancer was supported by a similar finding for duration of employment in the cotton industry, which did not rely on any assumptions about endotoxin concentrations. Sensitivity analyses revealed that increasing or decreasing the ‘opening’ mill area endotoxin measurements by a factor of two did not appreciably alter the results. Moreover, no similar association was apparent for the combination of cancers other than of the lung.

Ideally, our analysis would have taken into account the fact that part of each cohort member's work in the cotton industry and exposure to endotoxins occurred after his/her entry to follow-up. However, for the large majority of subjects, exposures after entry to follow-up were small in comparison with those accumulated earlier. Given the other approximations in the analysis, refinements to allow for continuing exposure during follow-up were not judged worthwhile. In support of this decision, the inverse relation between endotoxins and lung cancer remained when analysis was restricted to deaths occurring after 1987 (Figure 1), when all follow-up was after presumed last exposure.

Another possible source of error was healthy worker selection. Apart from any relation to cancer, cotton dust is a proven cause of the disabling respiratory disease, byssinosis. It is conceivable that the severity of byssinotic symptoms and disability is increased by smoking, and that as a consequence, workers who smoked were more likely to have left the cotton industry if they also had high cumulative exposure to cotton dust and therefore a greater risk of byssinosis. If so, the surviving population of workers with high exposure to cotton dust might have a lower prevalence of smoking than other cohort members, leading to spuriously low risks of lung cancer. Against this, however, the low risk of lung cancer with high exposure to endotoxins was observed after adjustment for smoking. Furthermore, after additional adjustment for baseline lung function and report of cough or phlegm, the trend was even stronger.

There is no reason why the relation between endotoxin exposure and subsequent mortality from lung cancer should be systematically different in the 10% of eligible subjects who declined to take part in the original surveys of respiratory disease, and therefore this incomplete participation would not be expected to bias comparisons of lung cancer risk according to endotoxin exposure. Similarly, the exclusion of workers of non-Caucasian ethnicity would not have biased comparisons of mortality internal to the cohort, although it limits the confidence with which findings can be generalised to non-Caucasian ethnic groups.

There is a possibility that when cohorts are followed towards maturity, incomplete ascertainment of deaths could lead to important underestimation of mortality rates at the oldest ages. To address this potential source of bias, we opted to censor all follow-up at age 90 years.

Mortality overall, and particularly from circulatory and respiratory disease, was higher than expected from national rates. Several factors may have contributed to this. First, part of the excess of respiratory disease was explained by 22 deaths from byssinosis – a direct consequence of work with cotton. There may also have been other deaths from byssinosis that were incorrectly ascribed to chronic obstructive pulmonary disease. Second, with such long follow-up, the lower-than-expected mortality from cardiovascular and respiratory disease that is normally seen in occupational cohorts because of a healthy worker effect tends to disappear. Third, the prevalence of smoking among female cohort members was relatively high (Table 2).

This high rate of smoking may also explain why despite their exposure to endotoxins, the overall SMR for lung cancer in women from the cohort was elevated (SMR 115, 95% CI: 86–148). However, when adjustment was made for smoking, a clear reduction in risk of lung cancer was seen with longer employment in the cotton industry and with higher cumulative exposure to endotoxins.

This observation is consistent with those from other cohort studies, which have indicated low rates of lung cancer in cotton textile workers overall, or in those with the highest or longest exposures to dust or endotoxins (Henderson and Enterline, 1973; Merchant and Ortmeyer, 1981; Hodgson and Jones, 1990; Szeszenia-Dabrowska *et al*, 1999; Wernli *et al*, 2003; Laakkonen *et al*, 2006; Kuzmickiene and Stukonis, 2007; Mastrangelo *et al*, 2008). The finding has not been universal (Buiatti *et al*, 1979; Koskela *et al*, 1990; Fritschi *et al*, 2004; Kuzmickiene *et al*, 2004), but it is supported by case–control studies (Lenters *et al*, 2010), and in a recent systematic review and meta-analysis, the summary relative risk of lung cancer in textile workers was 0.72 (95% CI: 0.57–0.90) (Lenters *et al*, 2010). Moreover, experimental studies in animals and limited trials in humans have indicated that endotoxins can induce antitumoural or cytotoxic responses (Reisser *et al*, 2002).

As well as adding to the evidence that endotoxins in cotton dust reduce the risk of lung cancer, our study suggests that the protective effect is restricted to lung tumours, depends on cumulative dose and persists after cessation of exposure – the low risk of lung cancer was clearly apparent in follow-up after 1987, at least 10 years after the large majority of cohort members would have last worked in the cotton industry. This pattern of findings would be compatible with a local protective mechanism in the lung acting at an early stage in carcinogenesis, or with the induction of an antitumour response, which once established continues even when there is no further exposure.

In light of these observations, there may be value in a study of biomarkers of immune and inflammatory response in the lungs of both current and former cotton mill workers when compared with unexposed controls.

## Figures and Tables

**Table 1:** Estimates of endotoxin concentrations in cotton mill working areas according to predominant type of fibre processed

**Fibre type**	**Mill area**	**No. of workers**	**Endotoxin concentration (EU** **m^−3^)**
Coarse or medium	Opening	335	18 000
	Carding	2394	18 000
	Ring room/winding	490	300
	Not known	101	—
			
Fine	Opening	2	9000
	Carding	22	9000
	Ring room/winding	37	150
	Not known	78	—

**Table 2:** Characteristics of subjects included in the analysis

**Characteristic**	**Men (*n*=1548)**	**Women (*n*=1911)**
*Age at 1 January 1966 (years)*	*Number (percentage) (%)*	*Number (percentage) (%)*
<20	266 (17)	102 (5)
20–29	219 (14)	196 (10)
30–39	278 (18)	406 (21)
40–49	293 (19)	536 (28)
50–59	365 (24)	568 (30)
60–69	118 (8)	94 (5)
>70	9 (1)	9 (0.5)
		
*Characteristics at recruitment*		
Smoking status		
Nonsmoker	333 (22)	856 (45)
Light smoker (1–14 cigarettes per day)	589 (38)	615 (32)
Medium/heavy smoker (⩾15 cigarettes per day)	536 (35)	391 (20)
Former smoker	90 (6)	49 (3)
		
Working area		
Unknown	47 (3)	132 (7)
Opening	329 (21)	8 (0)
Carding	1034 (67)	1382 (72)
Ring room/winding	138 (9)	389 (20)
		
Working hours		
Part time	14 (0.9)	248(13)
		
Cough or phlegm		
Present	508 (33)	493 (26)
		
FEV_1_ (% predicted)		
Mean (s.d.)	90 (19)	93 (19)
Range	10–168	15–146
		
*Time worked in cotton industry (years)*		
1–3	193 (12)	73 (4)
4–10	286 (18)	226 (12)
11–20	322 (21)	435 (23)
21–40	542 (35)	846 (44)
>40	205 (13)	331 (17)
		
*Status at end of follow-up*		
Lost to follow-up before age 90	44 (3)	44 (2)
Censored at age 90 and later lost to follow-up	0 (0)	2 (0.1)
Censored at age 90 and later died	40 (3)	101 (5)
Censored at age 90 and still alive	6 (0.4)	39 (2)
Died before age 90	900 (58)	1118 (59)
Alive and younger than age 90	558 (36)	607 (32)

Abbreviation: FEV_1_=forced expiratory volume of air in 1 second.

**Table 3:** Mortality of cohort by cause, 1966–2007

	**Men**			**Women**			**Both sexes**		
**Cause of death**	**No. of deaths**	**SMR**	**95% CI**	**No. of deaths**	**SMR**	**95% CI**	**No. of deaths**	**SMR**	**95% CI**
All causes	900	102	95–109	1118	107	101–113	2018	105	100–109
All MNs	223	92	81–105	292	106	94–118	515	99	91–108
All MNs excluding MN lung	149	94	80–110	238	104	91–117	387	100	90–110
MN nasopharynx	0	0	—	1	555	—	1	220	—
MN oesophagus	11	112	56–188	4	48	—	15	83	46–130
MN stomach	27	143	94–202	15	109	61–171	42	129	93–170
MN colon	9	56	25–99	30	128	86–177	39	99	70–132
MN rectum	15	163	91–256	12	141	72–232	27	152	100–215
MN liver	2	74	—	3	117	—	5	95	30–196
MN pancreas	15	148	83–232	11	85	42–143	26	113	74–160
MN larynx	2	95	—	2	262	—	4	139	—
MN lung	74	89	70–111	54	115	86–148	128	99	82–116
MN breast	0	0	—	36	68	47–91	36	67	47–91
MN ovary				16	89	51–139			
MN brain	3	67	—	6	133	48–261	9	100	45–177
MN thyroid gland	0	0	—	1	101	—	1	74	—
Circulatory system disease	431	104	94–114	535	116	106–126	966	110	103–117
Ischaemic heart disease	265	100	88–112	297	131	116–146	562	114	105–124
Cerebrovascular disease	96	121	98–147	143	105	88–123	239	111	97–125
Respiratory system disease	132	113	94–133	153	118	100–137	285	115	102–129
Bronchitis, emphysema and other COPD	55	97	73–125	57	149	113–190	112	118	97–141
Asthma	3	168	—	2	52	—	5	89	28–185
Byssinosis	15	92 879	51 823–145 829	7	22 410	8883–42 085	22	46 427	29 052–67 854

Abbreviations: CI=confidence interval; COPD=chronic obstructive pulmonary disease; MN=malignant neoplasm; SMR=standardised mortality ratio.

CIs not presented where there were <5 cases.

**Table 4:** Mortality from lung cancer during 1966 to 2007 by smoking habits and years worked in the cotton industry

		**Men**			**Women**			**Both sexes**		
**Smoking habits**	**Years worked in cotton industry**	**No. of deaths**	**SMR**	**95% CI**	**No. of deaths**	**SMR**	**95% CI**	**No. of deaths**	**SMR**	**95% CI**
⩾15 cigarettes per day	<15	10	217	103–372	7	433	171–813	17	273	159–418
	15–29	18	172	102–261	17	493	286–755	35	251	175–341
	⩾30	12	120	62–198	5	179	56–370	17	133	77–204
										
<15 cigarettes per day	<15	9	158	72–278	2	97	—	11	142	70–238
	15–29	9	72	33–127	13	202	107–327	22	116	73–170
	⩾30	12	84	43–138	7	123	49–230	19	95	57–143
										
Nonsmoker	<15	1	31	—	0	0	—	1	16	—
	15–29	1	14	—	1	11	—	2	13	—
	⩾30	0	0	—	2	17	—	2	10	—

Abbreviations: CI=confidence interval; SMR=standardised mortality ratio. CIs not presented where there were <5 cases.

Former smokers excluded because of small numbers (*n*=2 deaths).

**Table 5:** Modelled risk of death from lung cancer by smoking habits at recruitment and estimated cumulative exposure to endotoxins

	**Men**				**Women**				**Both sexes**			
	**HR**		**95% CI**	***N***	**HR**		**95% CI**	***N***	**HR**		**95% CI**	***N***
Nonsmoker	1			323	1			801	1			1124
<15 cigarettes per day	8.2		1.9–35.3	589	17.7		4.0–79.9	614	11.4		4.0–32.2	1203
⩾15 cigarettes per day	14.7		3.4–62.8	503	44.9		10.4–194.9	317	22.3		8.0–62.7	820
Former smoker	2.8		0. 4–20.4	86	NE			47	2.6		0.5–14.6	133
												
*Cumulative endotoxin exposure (EU m*^*−3*^ *years)*												
⩽30 000	1			157	1			400	1			557
>30 000 and ⩽200 000	1.3		0.5–3.1	445	0.6		0.2–1.7	269	0.8		0.5–1.6	714
>200 000 and ⩽400 000	0.9		0.4–2.0	357	0.9		0.4–1.8	370	0.7		0.4–1.3	727
>400 000 and ⩽600 000	0.8		0.4–1.9	298	0.5		0.2–1.2	336	0.6		0.3–1.0	634
>600 000	0.6		0.2–1.3	244	0.7		0.3–1.5	404	0.5		0.3–0.9	648
*P*-value for trend		0.05		*N*=1501		0.2		*N*=1779		0.005		*N*=3280

Abbreviations: HR=hazard ratio, *N*=number of cases; NE=not estimable; CI=confidence interval.

Analysis was based on 3280 subjects after exclusion of those (*n*=179) whose work area was unknown.

*P*-value for trend calculated using each individual subject’s exposure as a covariate in the model.
