# Supplementary material for: Lung cancer mortality in a cohort of UK cotton workers: an extended follow-up
Source: Br J Cancer. 2011 Aug 16;105(7):1054–60. doi: 10.1038/bjc.2011.312 (PMC3185933; doi:10.1038/bjc.2011.312)
Supplement: Supplementary Table [file bjc2011312x1.doc]

**Supplementary Table – Disease codes used in the British cotton study**

| Cause of Death | ICD Revision | | |
| --- | --- | --- | --- |
|  | ICD8:  1968-1978 | ICD9:  1979-2000 | ICD10:  2001 onwards |
| All MNs | 1400-2090 | 1400-2089 | C000-C979 |
| MN Nasopharynx | 1470 | 1470, 1471, 1472, 1473, 1478, 1479 | C111, C112, C119 |
| MN Oesophagus | 1500 | 1500, 1501, 1502, 1503, 1504, 1505, 1508, 1509 | C150, C151, C153, C154, C155, C158, C159 |
| MN Stomach | 1510, 1511, 1518, 1519 | 1510, 1511, 1512, 1513, 1514, 1515, 1516, 1518, 1519 | C160, C161, C162, C163, C164, C165, C166, C168, C169 |
| MN Colon | 1530, 1531, 1532, 1533, 1537, 1538, 1539 | 1530, 1531, 1532, 1533, 1534, 1535, 1536, 1537, 1538, 1539 | C180, C181, C182, C183, C184, C185, C186, C187, C188, C189 |
| MN Rectum | 1541 | 1541 | C200, C209 |
| MN Liver | 1550, 1551 | 1550, 1551,1552 | C220, C221, C222, C223, C224, C225, C226, C227, C228, C229 |
| MN Pancreas | 1570, 1578, 1579 | 1570,1571,1572,1573, 1574, 1578, 1579 | C250, C251, C252, C253, C254, C257, C258, C259 |
| MN Larynx | 1610, 1618, 1619 | 1610, 1611, 1612, 1613, 1618, 1619 | C320, C321, C322, C323, C328, C329 |
| MN Lung | 1620,1621 | 1620,1622,1623,1624, 1625,1628,1629 | C330, C339, C340, C341, C342, C343, C348, C349 |
| MN Breast | 1740 | 1740-1749 (female breast), 1750 (male breast) | C500, C506, C509 |
| MN Ovary | 1830 | 1830 | C560, C569 |
| MN Brain | 1910 | 1910, 1911, 1912, 1913, 1914, 1915, 1916, 1917, 1918, 1919 | C710, C711, C712, C713, C714, C715, C716, C717, C718, C719 |
| MN Thyroid gland | 1930 | 1930 | C730, C739 |
| Circulatory System Disease | 3900-4589 | 3900-4599 | I000-I999 |
| Ischemic Heart Disease | 4100-4149 | 4100-4149 | I200-I259 |
| Cerebrovascular disease | 4300-4389 | 4300-4380 | I600-I699 |
| Respiratory system diseases | 4600-5199 | 4600-5199 | J000-J999 |
| Bronchitis, emphysema and other COPD | 4900-4920, 4660, 5197 | 4900-4920, 4660, 4661, 4960 | J400-J449 |
| Asthma | 4930 | 4930, 4931, 4939 | J450, J451, J458, J459 |
| Byssinosis | 5161 | 5040 | J660, J668 |

MN = malignant neoplasm
